# Supplementary material for: Optimal exit choice during highway tunnel evacuations based on the fire locations
Source: PLoS One. 2021 Aug 20;16(8):e0256523. doi: 10.1371/journal.pone.0256523 (PMC8378735; doi:10.1371/journal.pone.0256523)
Supplement: Attachment — Submitted filename: Response to Reviewers.docx [file pone.0256523.s003.docx]

Response to Reviewers

Dear reviewers,

Thanks very much for taking the time to review this manuscript. I really appreciate all your comments and suggestions! Please find my itemized responses below and my revisions in the re-submitted files.

Thank you very much for your consideration.

Best regards!

Jianxiao Ma

**Reviewer #1:**

1. The authors used VISSIM to simulate the vehicle distributions when the fire happened. Please explain the advantage of VISSIM.

**Response:** Thanks for your suggestions. We have added papers 27 and 28 with more detail about the advantage of VISSIM in the Method section. The specific revises are as follows **(Word Lines: 153-156)**:

The car-following model of VISSIM could easily simulate and extract the trajectories of vehicles[27, 28]. Therefore, VISSIM is used to display vehicle distributions and operations in real time when the fire occurs [29], determining the locations of evacuating occupants and obstacles in the tunnel.

2. The research framework should be presented to indicate the methodology and contents.

**Response:** Thank you for your questions. We have added more descriptions to indicate the methodology and contents. The specific revises are as follows **(Word Lines: 78-87)**:

The aim in the study is to determine the optimal exit choice for different fire locations and evacuating occupants at different positions. We propose a strategy to obtain the optimal exit choice during highway tunnel evacuations based on fire location. The research framework includes three parts. First, we use BuildingEXODUS and VISSIM to obtain the parameters about vehicle distributions, locations of evacuating occupants, evacuation process and analyze the impacts of different fire locations on evacuation time. Then, the *OPS* output from BuildingEXODUS is selected as the evaluation index. Next, FCM cluster algorithm is used to capture the feature points of the occupants. Based on the feature points, the relationship between the location of fire and boundary of optimal exit choice under the optimal *OPS* is obtained through polynomial regression model.

3. The rows in Table 2 should be spaced equally.

**Response:** Thank you for your questions. In the revised version, we have modified Table 2**(Word Lines: 278)**.

4. The flowchart in Figure 4 of the paper is too cumbersome. The authors are advised to streamline it.

**Response:** Thank you for your questions. In the revised version, we have modified Figure 4.

5. The images inserted in the paper are blurry. It is recommended to use clear pictures.

**Response:** Thank you for your questions. In the revised version, we have provided figures of high quality.

6. The format of the references should be consistent. For example, the name of a paper with only reference 1 is capitalized.

**Response:** Thank you for your questions. In the revised version, we have modified the format of the references.

**Reviewer #2:**

1. It is not clear how parameters in VISSIM and BuildingEXODUS are calinbrated, and how the simulation results are validated. The whole calibration process for simulation based study is necessary and the author may want to inlcude more details in supporting information.

**Response:** Thank you for your questions. In the revised version, we have added more descriptions to expand the calibration process. The specific revises are as follows:

A set of simulation parameters is required to ensure the facticity of evacuation under the fire environment in the tunnel. In this study, monitoring data and literature data are collected to calibrate those simulation parameters input into BuildingEXODUS and VISSIM before simulation. Literature data are used to provide fire parameters and evacuation speed to ensure the authenticity of simulation. A previous emergency exercise of Maoshan Tunnel is used to validate the evacuation process in the highway tunnel under fire environment. **(Word Lines: 236-241)**

The parameters of fire determined based on both monitoring and literature data are input into the hazard module of BuildingEXODUS. **(Word Lines: 244-246)**

The maximum traffic volume is input into the VISSIM software to simulate the distribution of vehicles from freely flowing to congested traffic after the fire happens. **(Word Lines: 260-262)**

In this study, the average speed is 0.49 m/s, which is input into the movement module of BuildingEXODUS to simulate the movement of occupants in the highway tunnel under fire environment. **(Word Lines: 288-291)**

2. Only the optimal productivity statistics is used as the index for evaluating the evacuation efficiency. I believe there are many other indexes or metrics that may work in a similar way. The authors should discuss further on the reasoning behind the choice of the index and potentially include the results from other metrics.

**Response:** Thank you for your questions. There are some evaluation indexes used to evaluate evacuation efficiency, such as evacuation time. The calculation of *OPS* includes not only total evacuation time, but also the time of the last occupant evacuate from exit, which reflects the utilization rate and evacuation efficiency. From the perspective of management, the *OPS* could be used to measure the utilization rate of evacuation passageway and exit. In the revised version, we have added more descriptions to explain the reason for using the index. The specific revises are as follows **(Word Lines: 120-121)**:

The calculation of *OPS* includes not only total evacuation time, but also the time of the last occupant evacuating from exit, which reflects the utilization rate and evacuation efficiency.

3. The figures submitted are of very poor quality. This should be significantly improved. Vectorized images are preferred for scienific publications.

**Response:** Thank you for your questions. In the revised version, we have provided figures of high quality.

4. The current discussion on major findings is pretty slim. The authors should include more detailed discussion on how the results can inform better evacuation preparation in highway tunnels and what are the major lessons learned from the case study that can be carried over other scenarios. The authors should also discuss the limitations, which is important for simulation based studies.

**Response:** Thank you for your questions. In the revised version, we rewrote conclusion section. The specific revises are as follows **(Word Lines: 383-394)**:

Overall, the location of the fire is detected by fire detectors in the highway tunnel. The optimal exit choice during highway tunnel evacuations based on fire location is obtained by our strategy, which could provide valuable information for optimal exit choice and arranging variable evacuation signs in the highway tunnel. In our study, vehicle distribution is an important factor that determines the spatial location of vehicle and initial locations of evacuating occupants. Hence, effective information warning and traffic guidance could reduce the number of trapped vehicles and reduce accident casualties. In the future, we could study the optimal exit choice based on the fire locations, considering several evacuation passageways between the exit and entrance. Alternatively, we could study the impact of different fire parameters on the optimal exit choice of the highway tunnel.

**Editor:**

1. We suggest you thoroughly copyedit your manuscript for language usage, spelling, and grammar. If you do not know anyone who can help you do this, you may wish to consider employing a professional scientific editing service.

**Response:** We have employed a professional scientific editing service American Journal Experts（AJE）to provide language editing.

2. Please include captions for your Supporting Information files at the end of your manuscript, and update any in-text citations to match accordingly. Please see our Supporting Information guidelines for more information:

**Response:** We have added captions of Supporting Information files at the end of your manuscript and updated any in-text citations to match accordingly.

Special thanks to you for your good comments. We have carefully revised and polished the manuscript, please the reviewer to check.
